# The effectiveness of wet cupping vs. venesection on arterial O2 saturation level of cigarette smokers: A randomized controlled clinical trial

**DOI:** 10.12669/pjms.296.3365

**Published:** 2013

**Authors:** Hekmatpou D, Moeini L, Haji-Nadali S

**Affiliations:** 1 Hekmatpou D, PhD (Nursing), Assistant Professor, Faculty Member of Arak University of Medical Sciences, Arak, Iran.; 2 Dr. Moeini L, MD, Specialist in Pulmonary Diseases, Assistant Professor, Faculty Member of Arak University of Medical Sciences, Arak, Iran.; 3 Salar Haji-Nadali, Student of Nursing, Student Research Center Committee, Faculty Member of Arak University of Medical Sciences, Arak, Iran.

**Keywords:** Arterial O2 saturation, Cigarette smokers, Venesection, Wet cupping

## Abstract

***Objective:*** Wet cupping is a traditional bloodletting method recommended for controlling of respiratory disease complications. This study aimed to compare the efficacy of wet cupping vs. venesection on arterial O2 saturation level of smokers.

***Methods:*** This is a randomized controlled clinical trial which started with simple sampling of smokers. After administering spirometery, participants (*N* = 110 male smokers) with positive pulmonary function test (PFT), who manifested Chronic Obstructive Pulmonary Disease (COPD), were randomly assigned to intervention and control groups. The two groups were assessed in terms of demographic data, rate of hemoglobin (Hb), hematocrit (Hct), and arterial O2 saturation. Then, the intervention participants underwent wet cupping whereas venesection was performed on the control participants. At four stages after the two treatments, pulse oximetery was performed. Data was analyzed using SPSS (Version 17).

***Results: ***Result shows that mean arterial O2 sat level increased at three stages, namely before, immediately after, and 6 and 12 hrs after these two treatments (p ≤ 0.001). This indicates that wet cupping and venesection alike were effective on O2 sat level in the two groups, but the increasing pattern was maintained 12 hrs afterward only in those participants who had received wet cupping (p ≤ 0.001). Moreover, the results of repeated measure ANOVA between the two groups at the four stages showed that there were significant differences between the means of O2 saturation level at the 6- and 12-hrs stages (F = 66.92, p ≤ 0.001).

***Conclusion: ***Wet cupping caused a continued O2 saturation in the intervention group even up to 12 hrs afterward. Participants expressed liveliness and improved respiration after wet cupping. Therefore, wet cupping is recommended for promoting the health of cigarette smokers.

## INTRODUCTION

Cigarette smoking is one of the major problems of public health and premature deaths.^1^^,^^2^ Studies have shown that smoking is becoming increasingly prevalent in Asian countries compared to the rest of the world. Studies in central Iran reported that up to 15% of Iranians over age 19 smoked.^3^

About 90% of patients with a COPD are smokers. One of the effects of smoking is the increasing of hemoglobin, hematocrit, and red blood cell counts, which occur as a result of changes in blood gases.^4^

Despite great advances in medical sciences and overwhelming popularity of conventional medicine, the growth and development of public interest in and use of complementary or alternative medicine (CAM) are well documented.^5^

 Hijama means cupping, but in Arab and Muslim culture it refers to wet cupping.^6^ Wet cupping is over 5000 years old and it was possibly the Egyptians who first practiced bloodletting by scarification.^7^ The first precursors of cupping were Hippocrates and Galen, and after the advent of Islam, it was fully presented to humanity by Prophet Muhammad (PBUH).^8^ The mechanism of cupping therapy is not clear, but some researchers suggest that placement of cups on selected acupoints on the skin produces hyperemia or hemostasis, which results in a therapeutic effect.^9^^, ^^10^

Services and treatments provided by conventional medicine have proved costly for rehabilitated smokers. Besides, they are not totally satisfied with their treatment. These reasons and the gap in alternative medicine motivated the researchers of the present study to investigate the effects of wet cupping on arterial blood O2 saturation of smokers.

## METHODS

This study is a randomized controlled clinical trial, which is registered on IRICT.ir by 201108134519N2. Smokers visiting a lung clinic in Mahalat, central Iran, were chosen by simple method of sampling (sample size power = 80%, alpha = 0.05, size effect = 50% Altman nomogram for former and future studies = 49 patients). With a 5% probability of attrition, 55 subjects for each control and intervention groups were determined. The inclusion criteria were (a) granting formal consent, (b) not having acute respiratory illnesses or cold, (c) not having smoked for 3 - 6 hrs before, (d) and not having neuromuscular (e.g., myasthenia gravis) and metabolic (e.g. diabetes and blood) diseases. The exclusion criteria were (a) declaring disinclination halfway, (b) catching a cold, (c) smoking cigarettes during the study, (d)suffering from metabolic and hematologic diseases. After cigarette smokers were selected and agreed to participate voluntarily with written consent, they took a spirometery test.

Participants with a normal pulmonary function test were omitted and those with obstructive PFT (American Thoracic Society’s index of diagnosis) were randomly divided into two intervention and control groups. Then, the two groups were compared in terms of hemoglobin, hematocrit, and arterial O2 saturation using pulse oximetery (type *Critical Care 502*). Afshinjoo et al showed that there was a strong correlation between the percentage of arterial O2 saturation from pulse oximetery and the percentage of O2 saturation in arterial blood test (ABG) (*r* = 955%).^11^ Subsequently, wet cupping and venesection were performed on participants. Finally, 6 and 12 hrs after the intervention, pulse oximetry tests was performed. Data were analyzed using SPSS (Version 17).

In this study, wet cupping was carried out based on the common procedure. After a careful examination by a physician, if wet cupping is considered necessary and beneficial for him, the patient sits cross-legged on the bed. Then, the physician antisepticises a certain site using Betadine or alcohol and places a specially designed cup on it. After that, a suction device is used to vacuum the cup. The vacuum condition and the environmental air pressure pull the patient's skin into the cup so much so that it is firmly stuck to the skin. (The proper height of the skin dome from its top to the edge of the suction cup must be 1 to 1.5 cm.). This suction creates local congestion and inflammation, causing an accumulation of blood in the capillaries. After 3 to 5 minutes, the practitioner removes the cup and, utilizing a disposable sterile surgical razor, makes several scarifications, each 0.5 to 1 mm deep. The cup is put back on the same location and after re-suction; blood is gradually drawn out of the cuts. The cycle is repeated 3 to 5 times, each lasting for 3 to 5 min. In general, a wet cupping session takes 15 to 20 minutes. and 50 to 75 ml. blood in total is drawn out. In the control group, venesection was done using the sterile technique. The blood taken was 100 to 200 ml. The results of pulse oximetery at 6- and 12-hr stages after were compared in both groups.

***Ethical considerations:*** The study was a research project approved in Arak University of Medical Sciences, which was also confirmed by Clinical Research Ethics Committee of the University.

## RESULTS

The cohort of 110 smokers was randomly assigned to two groups, namely Wet Cupping (Hijama) and Venesection.

**Table-I:** Distribution of smokers based on demographic factors

*Variable*	*Type*	*Group*		*Statistical test X2/ P- value*
		*Wet Cupping (No/%)*	*Venesection (No/%)*	
				
Marriage Status	Married	46/83.6	40/72.7	1.91/0.16
	Single	6/16.4	15/27.3	
Education Level	Low Literates (Under Diploma)	38/69.1	37/67.3	8.5/ 0.075
	Diploma	14/25.5	8/14.5	
	University level	3/5.5	10/18.2	
Income*	Low	9/16.4	12/21.8	3.69/ 0.15
	Intermediate	33/60	23/41.8	
	Good	13/23.6	20/36.4	
Job	Worker	46/83.6	38/69.1	9.42/ 0.71
	Clerk	9/16.4	10/18.2	
	Retired	0/0	7/12.7	

*(Low=Income ≤ 250$/month, Intermediate= Income = 250- 500$/month, Good= Income ≥ 500 $/month)

Results of the statistical test of X^2^ did not show a significant difference between the two groups in terms of the above variables. The smoking variable was equal in both groups and all participants in the study (100%) had been smoking more than 10 years.

**Table-II:** Distribution of mean and standard deviation of special data of two groups

*Group*	*Wet cupping* *Mean ±SD*	*Venesection * *Mean ±SD*	*(p-value)*
*Variable*			
Age*	50.34 ± 6.7	50.45 ± 5.7	0.61
Weight (kg) **	80.8 ±10.21	79.6 ± 9.6	0.26
Height (cm) **	172 ± 6.6	173 ± 7	0 .28
Body Mass Index**	27.5± 3.7	26± 4	0 .71
Smoking duration **	14± 7.6	13 ± 6	0.65
Smoking no /day**	22± 5	23 ± 5	0 .75
Diagnosis of Disease**	4± 2.6 (year)	3.6 ± 2.5(year)	0.08

* t test, ** Man Whitney U test

The mean age of both groups was about 55 years. There were no significant differences in the distribution of participants in the two groups based on the variables mentioned. 

**Table-III:** Distribution of mean and standard deviation of other special data of two groups

*Group*	*Wet Cupping* *Mean ±SD*	*Venesection* *Mean ±SD*	*Man Whitney U* *(p-value)*
*Variable*			
Hemoglobin	17.7 ± 0.73	17.5 ±0.63	0.61
Hematocrit	53 ±1.7	54 ±1.6	0.26
FVC* rate	3.9 ±0.53	3.5 ±0.81	0.28
VC* rate	3.9 ±0.62	3.18 ±0.76	0.71
FEV1/FVC* rate	0.77 ±0.17	0.79 ±0.1	0.65
FVC%	0.81 ±0.17	0.79 ±0.15	0.75
VC%	3.5 ±3.5	3 ±4.1	0.08
FEV1/FVC%	0.78 ±0.17	0.79 ±0.16	0.35

**FVC** ** rate = Force Vital Capacity **

**VC** ** rate = Vital Capacity **

**FEV1/FVC** ** rate = Force Expiratory Volume in the first second/ Force Vital Capacity**

Results from the Mann-Whitney U test do not display any significant difference between the two groups before intervention. In other words, the distribution of participants in the two groups did not have any significant difference in terms of variables mentioned.

**Table-IV:** Distribution of mean and standard deviation of arterial O2 Saturation of two groups

*Group*	*Wet Cupping* *Mean ±SD*	*Venesection* *Mean ±SD*	*t test* *(p-value)*
*Variable*			
O2Sat. Before	88.5 ±3.7	88.5 ±4.1	0.75
O2Sat. Immediately after	89 ±3.6	89 ±4.2	0.88
O2Sat. 6 hours after	93.5 ±3	92.5 ±3	≤ 0.001
O2Sat. 12 hours after	94 ±3.3	92 ±3.4	≤ 0.001

Results show that in both groups, arterial O2 saturation rose at all four stages that is before, immediately after, six hrs after and 12 hrs after wet cupping and venesection. That is to say both venesection and wet cupping were effective on O2 sat. Results of a paired t-test showed that the mean arterial O2 saturation before, after, six and twelve hrs after wet cupping were significantly different (p ≥ .001); that is, the arterial O2 saturation at the four stages was on the rise. Although the results of the paired t-test of the venesection group showed that the mean arterial O2 saturation at all the four stages were significantly different (001/0 ≥ P), this increasing trend stopped after the 12-hr stage. There was no significant difference between the mean arterial O2 saturation levels six and twelve hrs after venesection (p ≥ .035).

**Fig.1:**
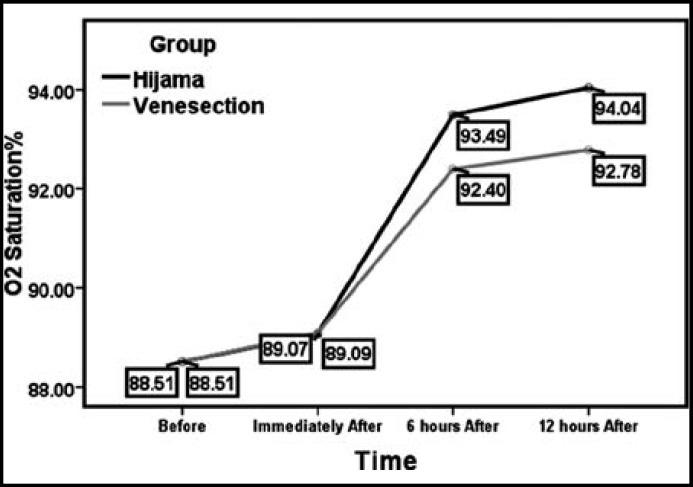
Differences among O2 Sat.Rate in two groups

Doing repeated measure ANOVA test between the two groups at four stages (Wilks' Lambda), indicates a significant difference between the mean arterial O2 saturation between the two groups 6 and 12 hrs after the two measures (p ≥ .001 and F = 66.92).

## DISCUSSION

Based on wet cupping hypothesis, wet cupping bloodletting is different from venous bloodletting in that the blood is drawn from capillary tubes and includes some lymph fluid, modifying its concentration and eliminating waste materials.^8^^, ^^9^ Between the two shoulders, lymphatic and venous capillary systems are built such that toxins of blood, heavy particles, and harmful wandering macro-elements are caught in the blood vessels. Wet cupping in this area of the body sucks out contaminated blood.^8^

In this study the results indicated that in both groups arterial O2 saturation at the four stages were on the rise. This means that both interventions, i.e. wet cupping and venesection, were effective on O2 saturation. The means of arterial O2 saturation at before, after, six, and twelve hrs stages of wet cupping were significantly different (p ≥ .0001), meaning that the saturation of arterial O2 at the four stages was on the rise. As for the Venesection group, there were significant differences between the means of arterial O2 saturation at the four stages (p ≥ .001). However, this growing trend stopped 12 hrs afterward, and there was no significant difference between the mean arterial blood O2 saturation at 6- and 12- hr stages (p ≥ .035). Apparently, this difference, especially after the 12-hr stage of wet cupping, is due to the difference in the biochemical composition of blood. A study comparing the venous and wet cupping bloods showed that the amount of LDL and TG and the levels of RBC, Hb, hematocrit, and blood viscosity were higher in the wet cupping blood.^12^^, ^^13^

In another study, it was reported that wet cupping lead to a significant increase of HDL levels and reduction of LDL and triglycerides.^14^ 15. Thomas and Wilson in their study have stated that Bernard et al demonstrated the effect of wet cupping on O2 uptake increase in patients with polycythemia.^15^ A comparison of venous and wet cupping blood showed that the concentration of uric acid, urea, triglycerides, and cholesterol was significantly higher in the wet cupping blood.^16^ Shekarforoush and Foadoddini in their study have stated that the rate of ischemic induced arrhythmias was significantly modified by wet cupping (P < 0.05). These results indicate for the first time in rats that cupping might be cardioprotective in the ischemic reperfusion injury model.^17^

ANOVA test results between the Wet cupping and Venesection Groups at the four stages (Wilks' Lambda), indicated a significant difference between the mean arterial O2 saturation in both groups at the 6- and 12-hr stages (p ≥ 0.001 and F = 66.92). This discrepancy can be due to viscosity reduction following the wet cupping. Because comparatively more amounts of lipids, uric acid, and other waste material are removed from blood after wet cupping, arterial O2 saturation would be higher after wet cupping. In this regard, several studies have been conducted which show different blood density in wet cupping and venesection bloods.

 In Fazel et al. study the results showed that LDL levels after wet cupping decreased significantly (p > .001). Changes in the LDL / HDL ratio using the *t*-test showed that this ratio had declined more in the wet cupping group than the control group, and that there was a significant difference between the two groups (p > .004). Hence, by reducing LDL and the LDL / HDL ratio, wet cupping can play a pivotal role in eliminating the risk factors of atherosclerosis and cardiovascular diseases.^16^ Daniali et al in their study have stated that the amount of uric acid, HDL, TG, LDL, SGOT and iron levels were higher in the wet cupping blood, and this difference vs. venous bloods was statistically significant (p < .001). Results also showed that the amount of red blood cells, hemoglobin, hematocrit, viscosity, MCHD (Mean Corpuscular Hemoglobin Concentration) in the wet cupping blood compared with venous blood were significantly higher (p < .013).^18^ In one study the results suggested significant effect in favor of cupping on vascular compliance and degree of vascular filling.^19^

These results show that venous blood and wet cupping blood are different in terms of biochemical properties. This can be due to the area on which wet cupping and phlebotomy (venesection) were done and differences that can be associated with the adverse site or manner of wet cupping. It seems that these differences could increase the arterial O_2 _saturation rate among smokers, but for having better advantages doing clinical trials with more samples is recommended.

## CONCLUSION

This study provided evidence that performing wet cupping on cigarette smokers leads to enhanced arterial O2 saturation with an increasing trend 12 hours afterward. Those participants who had received the wet cupping treatment expressed satisfaction on better breathing. Therefore, wet cupping is recommended for promoting the health of cigarette smokers.
